# Comparative ultrastructure of the antennae and sensory hairs in six species of crayfish

**DOI:** 10.7717/peerj.15006

**Published:** 2023-03-07

**Authors:** Golara Kor, Kifayatullah Mengal, Miloš Buřič, Pavel Kozák, Hamid Niksirat

**Affiliations:** Faculty of Fisheries and Protection of Waters, South Bohemian Research Centre of Aquaculture and Biodiversity of Hydrocenoses, University of South Bohemia in České Budějovice, Vodňany, Czech Republic

**Keywords:** Antenna, Arthropods, Biometry, Electron microscopy, Morphology, Crustaceans

## Abstract

**Background:**

Antennae in crayfish are essential for gaining information about the local topography and localising food, chemicals, conspecifics or predator. There are still gaps in the research on the morphology of antennae in decapods compared to other arthropods.

**Methodology:**

Biometrical and ultrastructural methods were applied using light and cryo-scanning electron microscopies to study the morphology of antennae in six different crayfish species, including marbled crayfish *Procambarus virginalis*, Mexican dwarf crayfish *Cambarellus patzcuarensis*, red swamp crayfish *Procambarus clarkii*, signal crayfish *Pacifastacus leniusculus*, common yabby *Cherax destructor*, and spiny-cheek crayfish *Faxonius limosus* to find their potential morphological differences.

**Results:**

Significant differences in the antenna length, length and width of each segment to carapace length ratios, and the number of segments were found in the six crayfish species. The ultrastructure revealed differences in the distribution of sensory hairs on the antenna and the morphology of the antennal surface.

**Conclusions:**

The different morphology of antennae might reflect adaptation to the conditions of their specific habitats. In addition, results showed that a combination of differences in the morphological features and biometrical measurements of antennae could be used for the distinguishment of different studied crayfish species.

## Introduction

Decapod crustaceans bear segmented sensory organs on the anterior part of their bodies called antennae (Mellon, 2012). They are well-equipped with such sensory appendages that allow them to sense chemical and hydrodynamic stimuli in their environments (Pravin et al., 2015). Such information from the antennae is used for several purposes, including detection and identification of predators, potential mates, gaining tactile information about the local topography (Basil & Sandeman, 2000; Zeil, Sandeman & Sandeman, 1985), localising food, or communicating and interacting with conspecifics (Pravin et al., 2015; Sandeman, 1985). A study of antenna morphology may also enhance our knowledge about the taxonomic and ecological status of target animals (Hao, Sun & Liu, 2020). An earlier study stated that the morphology of the setae in the antennule may reflect the phylogeny of freshwater crayfish (Hobbs, 1974). On the other hand, it can improve our understanding of the potential differences in sensory ability among different species of crayfish and provide detailed information about the selection of the proper model animal for future behavioural and toxicological studies.

Crayfish are a diverse group of decapods that are distributed across the different freshwater ecosystems in the world (Breithaupt & Eger, 2002; Holdich & Lowery, 1988; Vogt, 2002). Currently, about 700 species of these animals are identified across different kinds of freshwater ecosystems, and several new species are listed each year (Crandall & De Grave, 2017). Freshwater crayfish have important ecological roles and impacts as native and invasive species in freshwater ecosystems (Patoka, Kalous & Kopecký, 2014; Patoka, Kocánová & Kalous, 2016).

The antennae of other Arthropoda, such as insects, have been the subject of many morphological studies, including Diptera (Zhang et al., 2021), Lepidoptera (Limberger, Brugnera & da Fonseca, 2021), Coleoptera (Hao, Sun & Liu, 2020), Hymenoptera (Yang et al., 2021) *etc*., but there is scarce information regarding the fine structure of this segmented appendage in crayfish (Bender, Gnatzy & Tautz, 1984; Masters et al., 1982; Sandeman, 1985; Sandeman, 1989).

Therefore, we applied light and cryo-scanning electron microscopies to investigate the biometrical and ultrastructural features of the antennae from six crayfish species inhabiting different habitats, including marbled crayfish *Procambarus virginalis*, Mexican dwarf crayfish *Cambarellus patzcuarensis*, red swamp crayfish *Procambarus clarkii*, spiny-cheek crayfish *Faxonius limosus* (Cambaridae), signal crayfish *Pacifastacus leniusculus* (Astacidae) and common yabby *Cherax destructo*r (Parastacidae) to find the potential morphological differences among species.

## Materials and Methods

### Animals

All protocols and ethics for animal handling were approved by the national laws and regulations on animal welfare 246/1992, and the institutional animal care guidelines of the Faculty of Fisheries and Protection of Waters of the University of South Bohemia in České Budějovice. All efforts were made to reduce animal suffering. It should be noted that no animals were killed during our study.

Marbled crayfish (19.7 ± 0.3 mm, *n* = 10), red swamp crayfish (41.2 ± 1.8 mm, *n* = 10), spiny-cheek crayfish (24.5 ± 1.7 mm, *n* = 8), and common yabby (27.1 ± 1.6 mm, *n* = 6), crayfish were obtained from the experimental culture of the Laboratory of freshwater ecosystems, Faculty of fisheries and protection of waters, Vodňany, Czech Republic. Mexican dwarf crayfish (8.5 ± 0.2 mm, *n* = 10) were purchased from the pet shop. Signal crayfish (42.7 ± 0.7 mm, *n* = 10) were caught from Křesánovský creek near Vimperk, Czech Republic. Crayfish were separately acclimatised in plastic boxes with 1 L of aerated aged tap water before the experiment. Prior to sampling, the animals were anaesthetised on ice for 10 min. Antenna samples were collected equally from both sexes of adult crayfish using scissors and used for biometrical and ultrastructural purposes.

### Biometrical measurements

Intact animals without injuries were used for the experiment. The light microscope (Olympus SZX16; Olympus, Shinjuku City, Japan) equipped with a DP74 camera was applied. The carapace length, antenna length, length and width of each antennal segment were measured by ImageJ (NIH, Bethesda, MD, USA). Antenna total length, width and lengths of each antennal segment in the different species were adjusted by dividing into the carapace length to remove differences among the size of animals (Weiss, Thatje & Heilmayer, 2010).

For statistical analysis, Kolmogorov–Smirnov and Levene’s tests were used for the normality and homogeneity of variance using Statistica 13 software (StatSoft, Inc., Tulsa, OK, USA). For normal data (antenna to carapace length ratio), the one-way analysis of variance (ANOVA) with subsequent Tukey’s *post-hoc* test was carried out to evaluate differences among species. The length and width of each segment to carapace ratios, and number of segments were not normally distributed and assessed using the non-parametric Kruskal-Wallis test with subsequent multiple comparisons *post-hoc* statistical analysis. For all statistical tests, *p* < 0.05 was considered significant. Data are expressed as the mean ± SEM.

### Electron microscopy

Scanning electron microscopy (SEM) JEOL 7401F (JEOL Ltd., Akishima, Japan) equipped with a cryo-attachment CryoALTO 2500 (Gatan, Inc., Pleasanton, CA, USA) was used to study the antennal ultrastructure and sensory hairs. The samples were placed on the stub using double copper tape and Tissue-Tek. The samples were frozen by plunging into a liquid nitrogen slush. After freezing, the samples were transferred to the high vacuum cryo-preparation chamber, where the surface of the samples were sublimated at a temperature of −98 °C for 2 min. After sublimation, the samples were coated with 5 nm of gold at −135 °C. The coated samples were inserted into the Field Emission Electron Microscope (JSM-7401-F; JEOL Ltd., Akishima, Japan) precooled at −135 °C. Images were obtained using an Everhart-Thornley detector at an accelerating voltage of 1.5 kV using Gentle Beam (GB) low mode (Niksirat, Kouba & Kozak, 2015).

## Results

### Biometrical measurements

**Figure 1:**
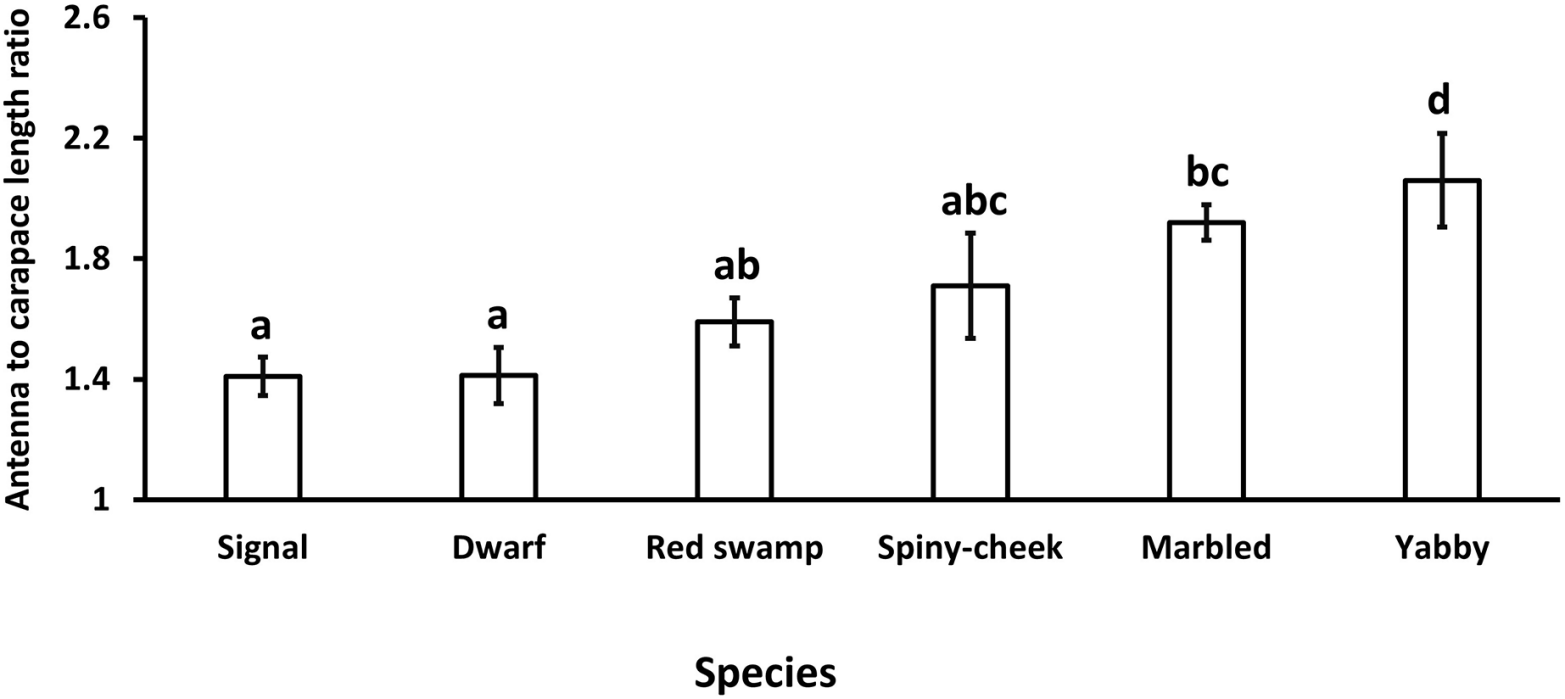
Comparison of antenna to carapace length ratio among six species of freshwater crayfish, including marbled crayfish *Procambarus virginalis*, Mexican dwarf crayfish *Cambarellus patzcuarensis*, red swamp crayfish *Procambarus clarkii*, signal crayfish *Pacifastacus leniusculus*, common yabby *Cherax destructor*, and spiny-cheek crayfish *Faxonius limosus*. Letters indicate significant differences between species.

Our results showed a significant (*p* = 0.0005, df = 5) difference in the antenna to carapace length ratio among our studied species. While common yabby showed the longest antenna to carapace length ratio, the signal and Mexican dwarf crayfish had the lowest values of this ratio (Fig. 1).

**Figure 2:**
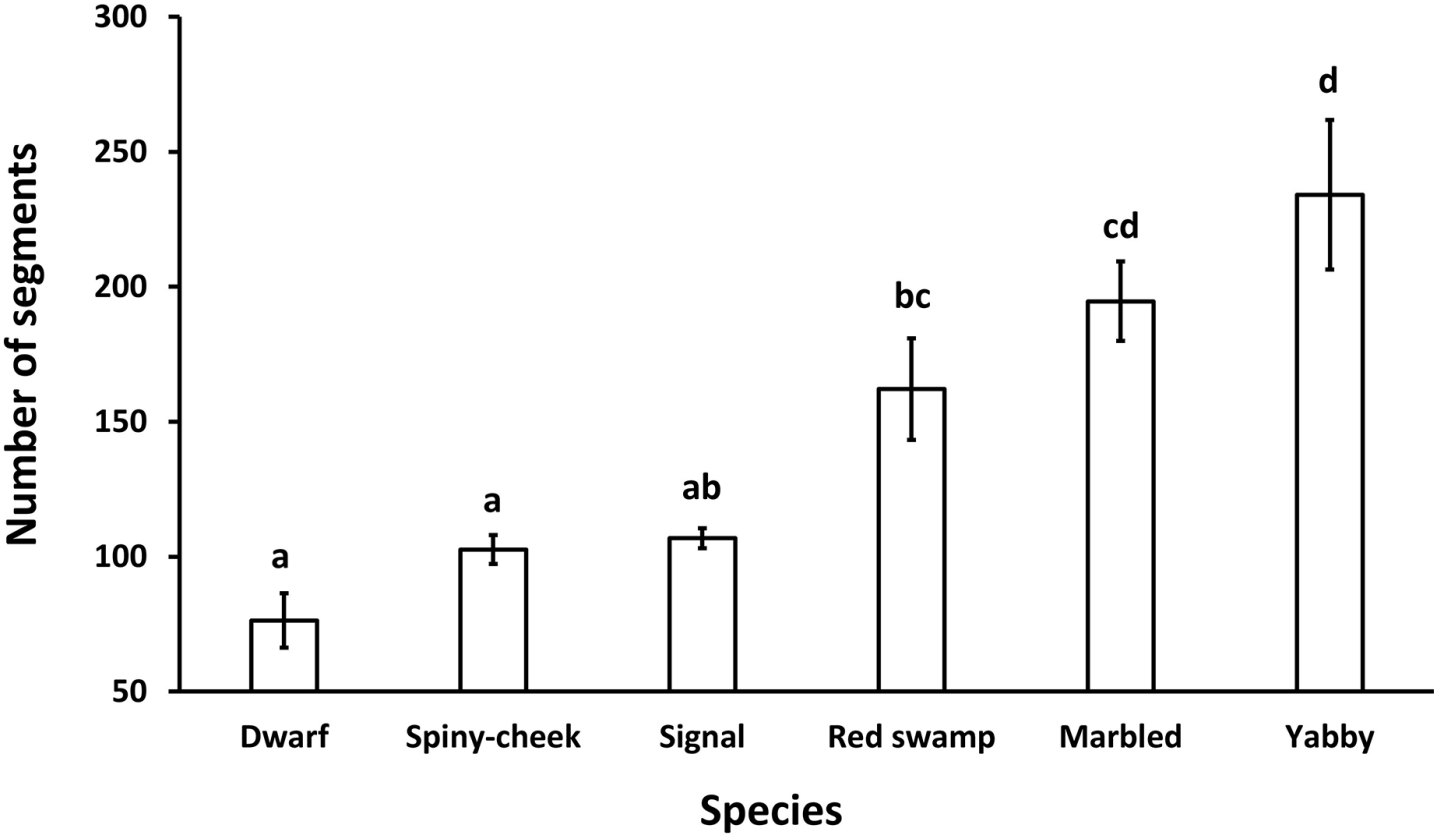
Comparison of the number of segments in the antenna of six freshwater crayfish species including marbled crayfish *Procambarus virginalis*, Mexican dwarf crayfish *Cambarellus patzcuarensis*, red swamp crayfish *Procambarus clarkii*, signal crayfish *Pacifastacus leniusculus*, common yabby *Cherax destructor*, and spiny-cheek crayfish *Faxonius limosus*. Letters indicate significant differences between species.

The results showed a significant (*p* = 0.0001, df = 5) difference among the number of segments of the antennae in different species. While common yabby showed the highest number of segments, the Mexican dwarf and spiny-cheek crayfish had the lowest number of segments (Fig. 2).

The results indicated a significant (*p* = 0.0001, df = 5) difference among the lengths of each antenna segment to carapace length ratio in the different species. The longest and shortest lengths of the lower, middle and upper parts of each antenna segment were recorded in Mexican dwarf and red swamp crayfish, respectively (Table 1).

**Table 1:** Comparison of different length of each antenna segment/carapace length ratio in the different species of freshwater crayfish.

	Spiny cheek	Dwarf	Signal	Red swamp	Marbled	Yabby
Upper	a(B) 0.0095 ± 0.0002	b(B) 0.0167 ± 0.0003	c(B) 0.0125 ± 0.0002	d(C) 0.0068 ± 0.0001	a(C) 0.0102 ± 0.0001	a(B) 0.0098 ± 0.0002
Middle	a(A) 0.0106 ± 0.0002	b(AB) 0.0170 ± 0.0003	b(A) 0.0145 ± 0.0001	c(B) 0.0076 ± 0.0001	a(B) 0.0109 ± 0.0001	a(B) 0.0097 ± 0.0001
Lower	a(A) 0.0121 ± 0.0001	b(A) 0.0174 ± 0.0003	c(A) 0.0138 ± 0.0002	d(A) 0.0081 ± 0.0001	a(A) 0.0119 ± 0.0001	d(A) 0.0085 ± 0.0001
Total	a 0.0105 ± 0.0001	b 0.0170 ± 0.0001	c 0.0136 ± 0.0001	d 0.0075 ± 0.00006	a 0.0110 ± 0.0001	e 0.094 ± 0.0001

**Note:**

Within columns and rows, values marked with different capital and small letters indicate significant differences, respectively.

Our results showed a significant (*p* = 0.0001, df = 5) difference among the widths of each antenna segment to carapace length ratio in the studied species. The width of each segment for all species decreased from the lower to the upper parts of the antenna. For the different parts of the antenna, the widest and narrowest were recorded in Mexican dwarf and marbled crayfish in the upper part, common yabby and marbled crayfish in the middle part, and signal and Mexican dwarf crayfish in the lower part, respectively (Table 2).

**Table 2:** Comparison of different width of each antenna segment/carapace length ratio in the different species of freshwater crayfish.

	Spiny cheek	Dwarf	Signal	Red swamp	Marbled	Yabby
Upper	a(C) 0.0081 ± 0.0001	a(C) 0.0091 ± 0.0007	bc(C) 0.0079 ± 0.0001	ac(C) 0.0074 ± 0.0001	b(C) 0.0065 ± 0.0001	a(C) 0.0083 ± 0.0002
Middle	ab(B) 0.0126 ± 0.0002	b(B) 0.0134 ± 0.0002	c(B) 0.0156 ± 0.0002	c(B) 0.0148 ± 0.0001	a(B) 0.0124 ± 0.0001	d(B) 0.0169 ± 0.0002
Lower	a(A) 0.0219 ± 0.0003	a(A) 0.0208 ± 0.0002	b(A) 0.0266 ± 0.0002	a(A) 0.0221 ± 0.0002	a(A) 0.0229 ± 0.0003	b(A) 0.0260 ± 0.0003
Total	a 0.0144 ± 0.0003	ab 0.0148 ± 0.0003	b–e 0.0164 ± 0.0003	ae 0.0148 ± 0.0003	a 0.0140 ± 0.0003	d 0.0171 ± 0.0003

**Note:**

Within columns and rows, values marked with different capital and small letters indicate significant differences, respectively.

### Ultrastructural features

**Figure 3:**
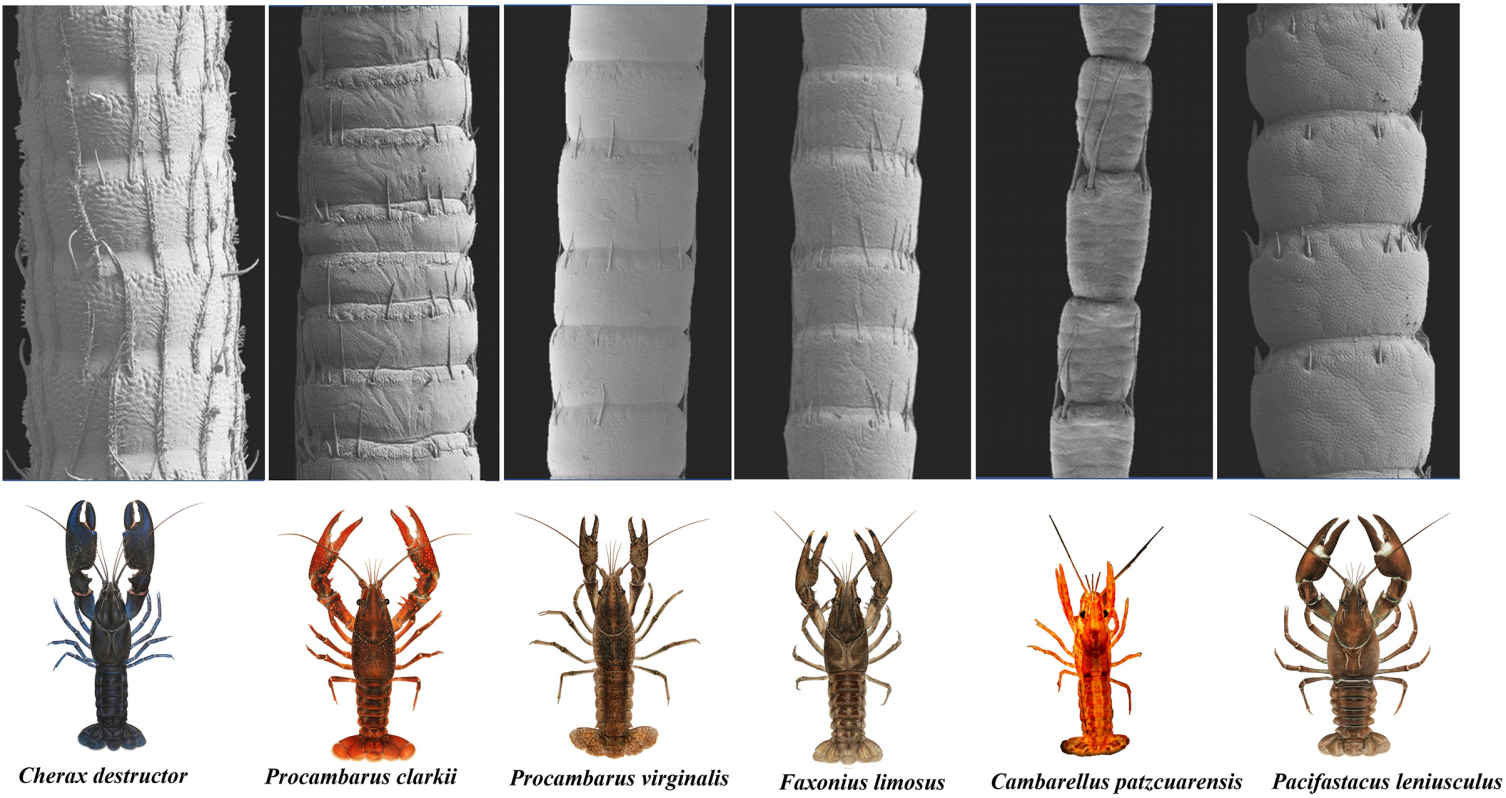
A general view of the morphology of antennae in six species of crayfish.

**Figure 4:**
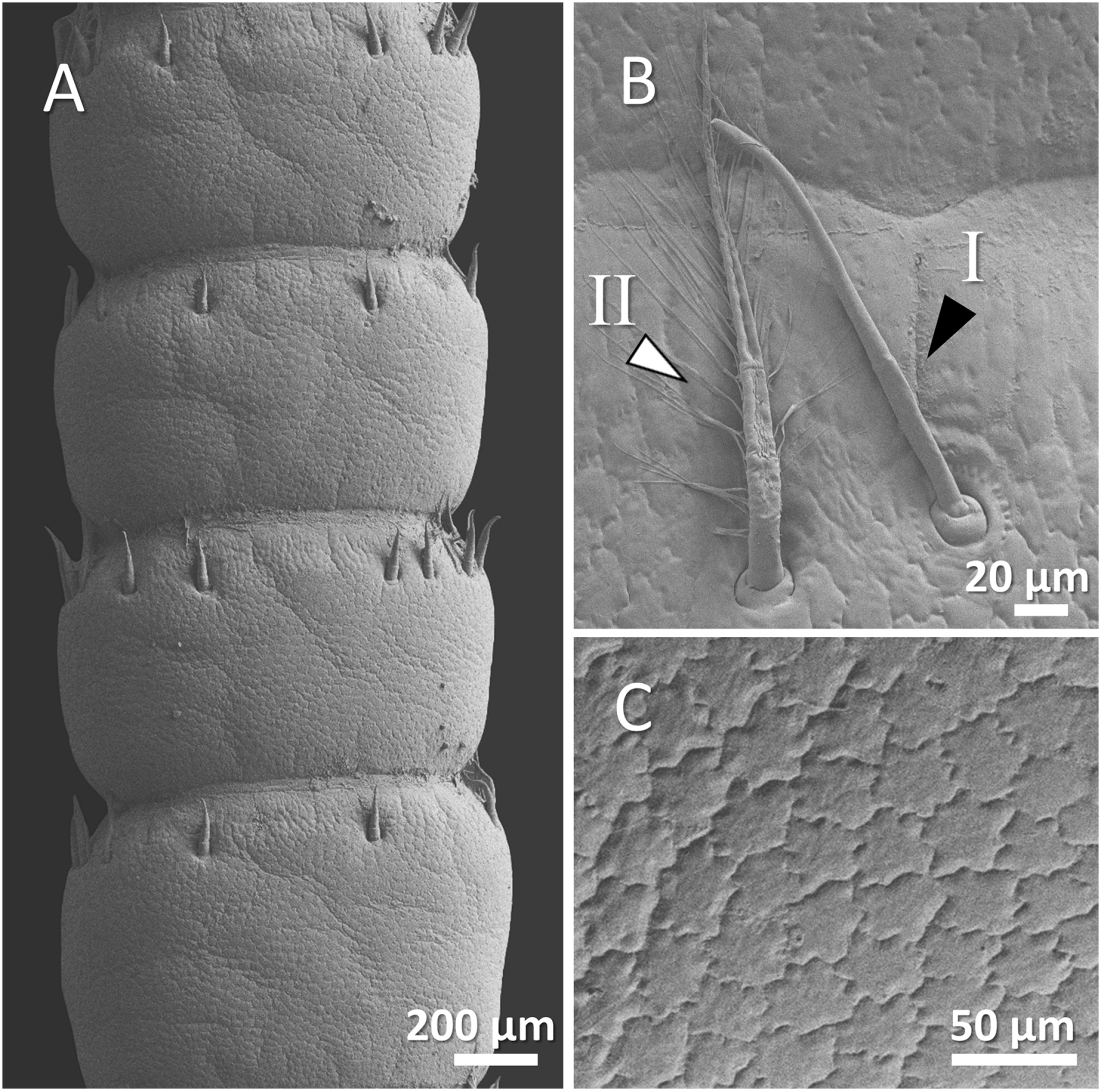
(A) An overview of antenna in signal crayfish (*Pacifastacus leniusculus*), (B) a higher magnification of two forms of sensory hairs (I, non-branched; II, branched), (C) a higher magnification of squamous surface of antenna.

**Figure 5:**
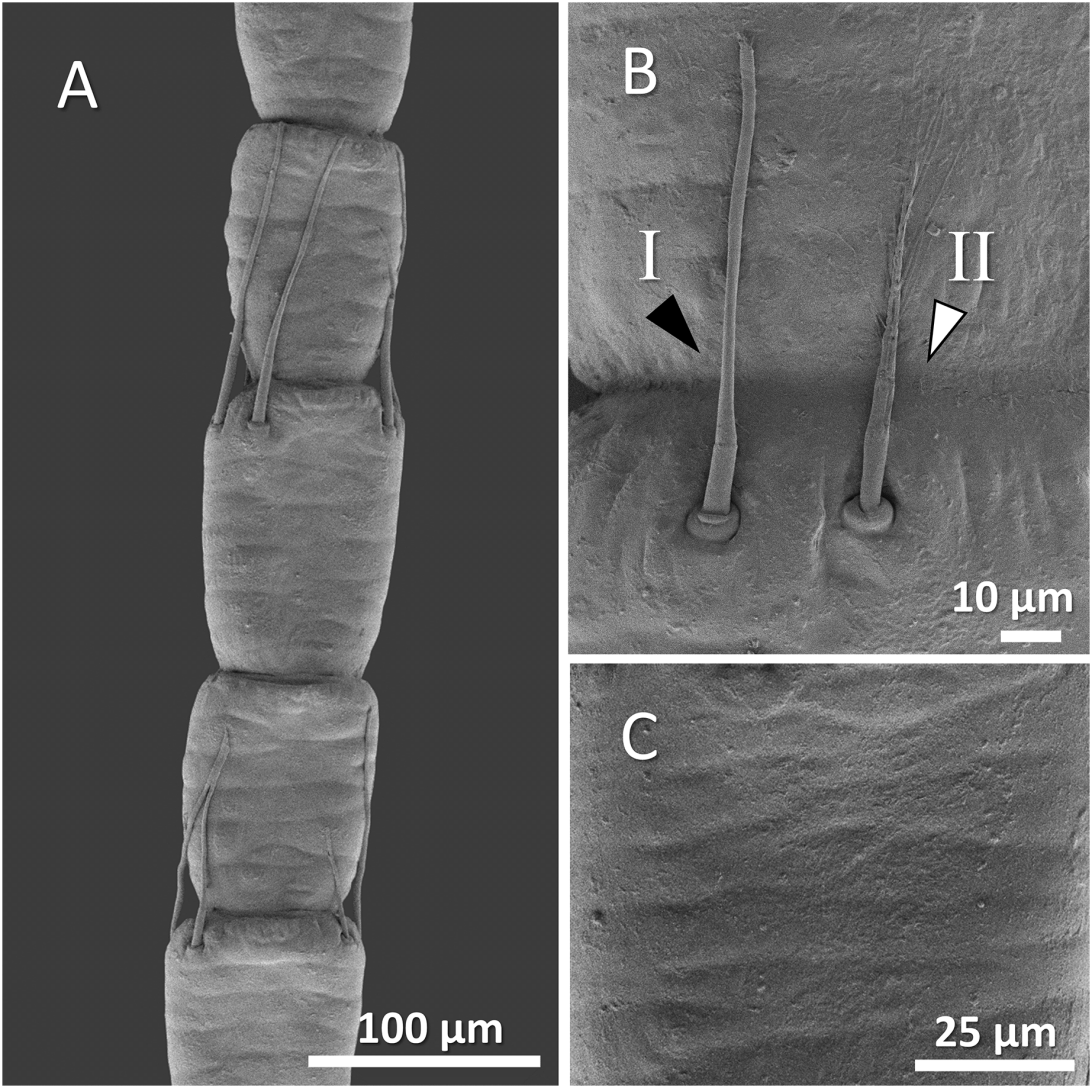
(A) An overview of antenna in Mexican dwarf crayfish (*Cambarellus patzcuarensis*), (B) a higher magnification of two forms of sensory hair (I, non-branched; II, branched), and (C) a higher magnification of wavy and wrinkled surface of antenna.

**Figure 6:**
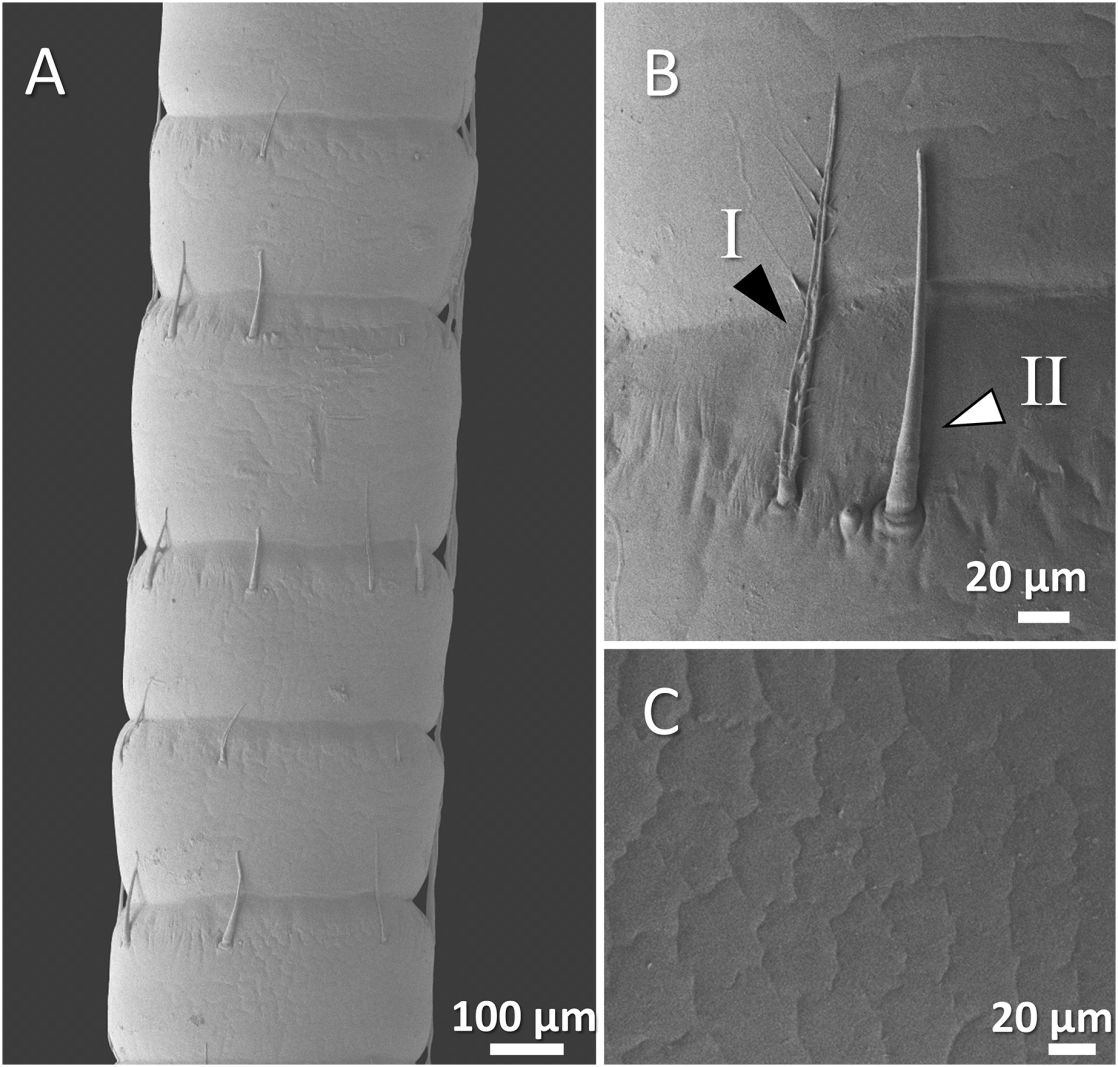
(A) An overview of antenna in marbled crayfish (*Procambarus virginalis*), (B) a higher magnification of two forms of sensory hair (I, branched; II, non-branched), (B and C) a higher magnification of relatively squamous surface of antenna.

**Figure 7:**
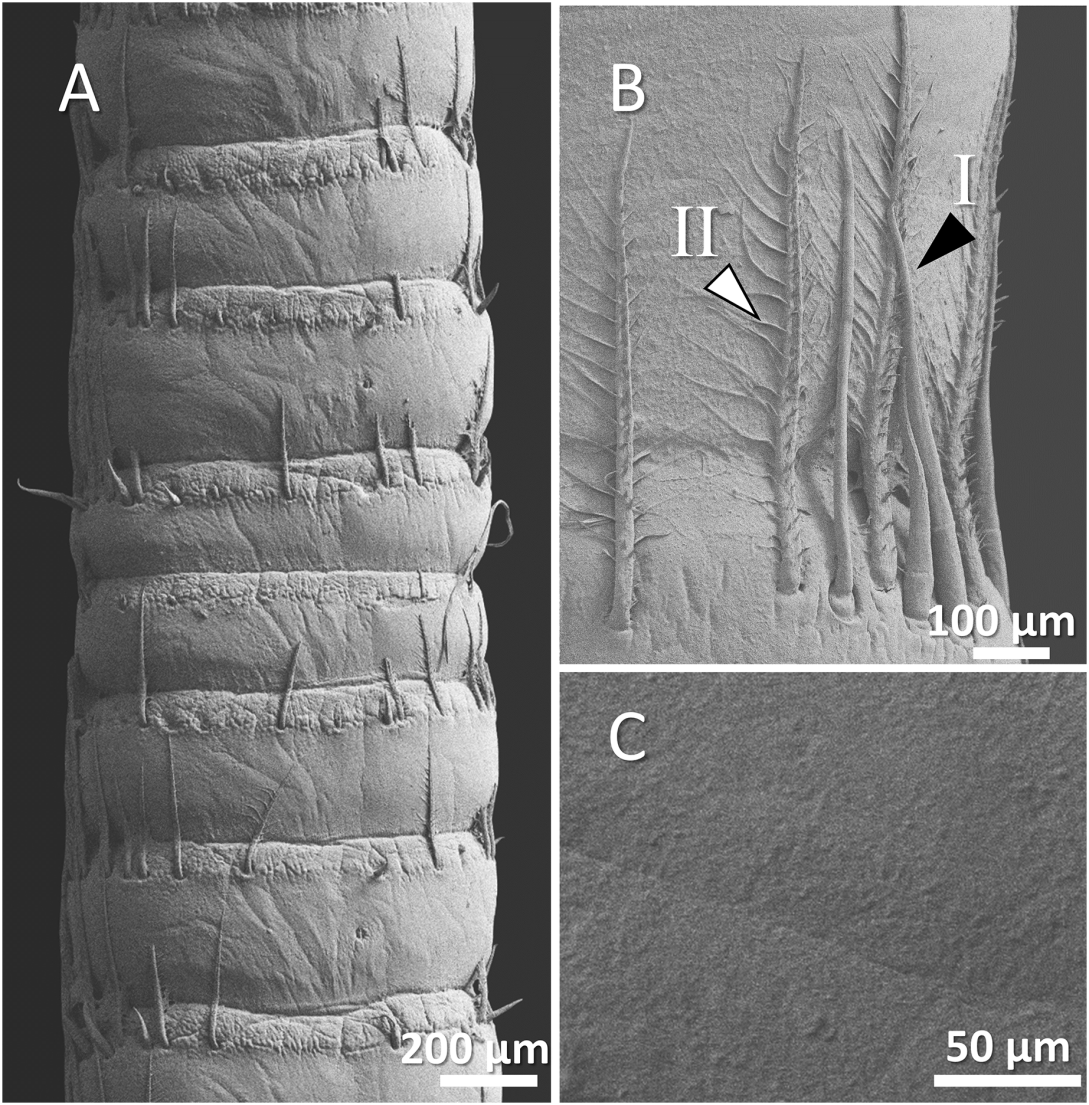
(A) An overview of antenna in red swamp crayfish (*Procambarus clarkii*), (B) a higher magnification of two forms of sensory hair (I, non-branched; II, branched), and (C) a higher magnification of relatively smooth surface of antenna.

**Figure 8:**
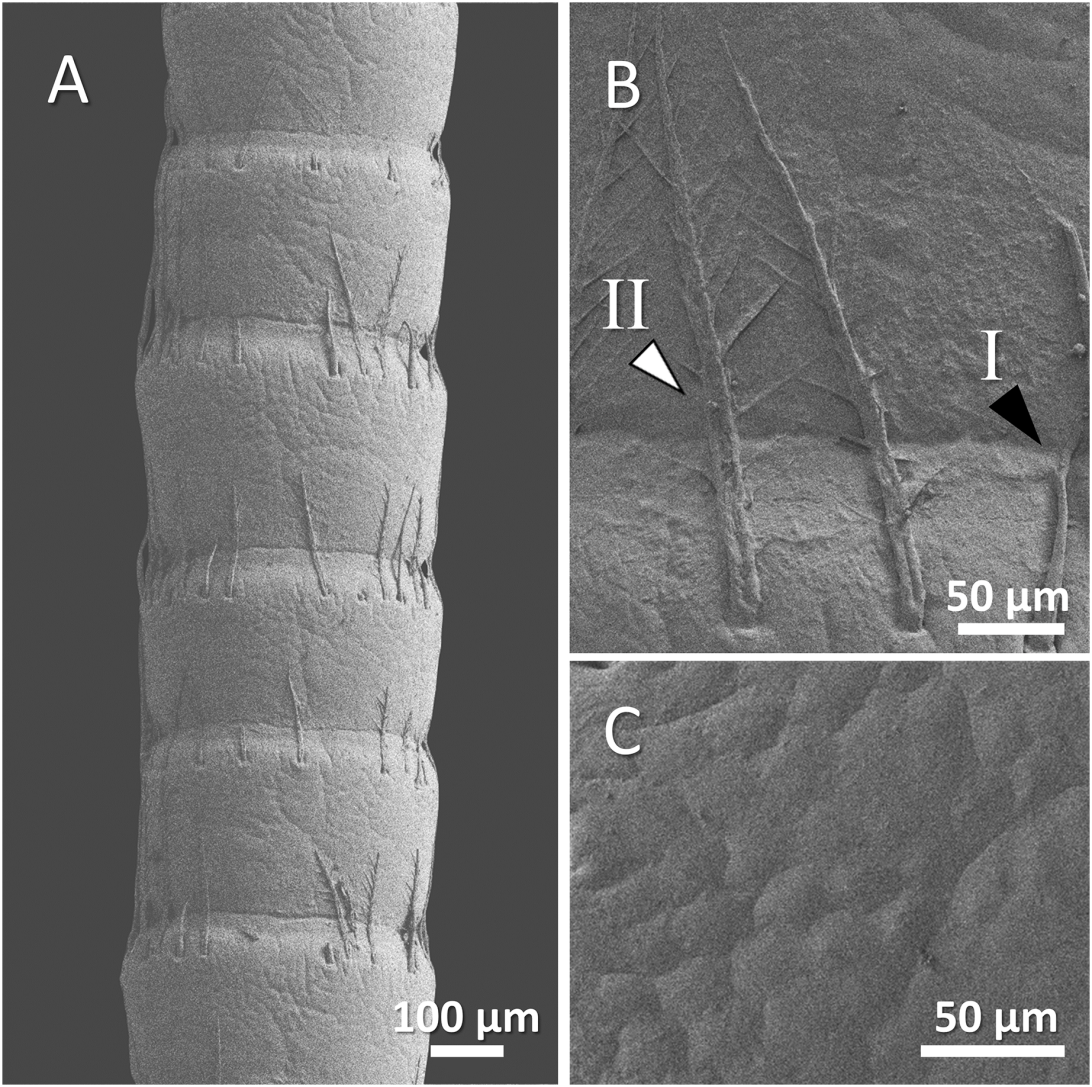
(A) An overview of antenna in spiny-cheek crayfish (*Faxonius limosus*), (B) a higher magnification of two forms of sensory hair (I, non-branched; II, branched), and (C) a higher magnification of relatively squamous surface of antenna.

**Figure 9:**
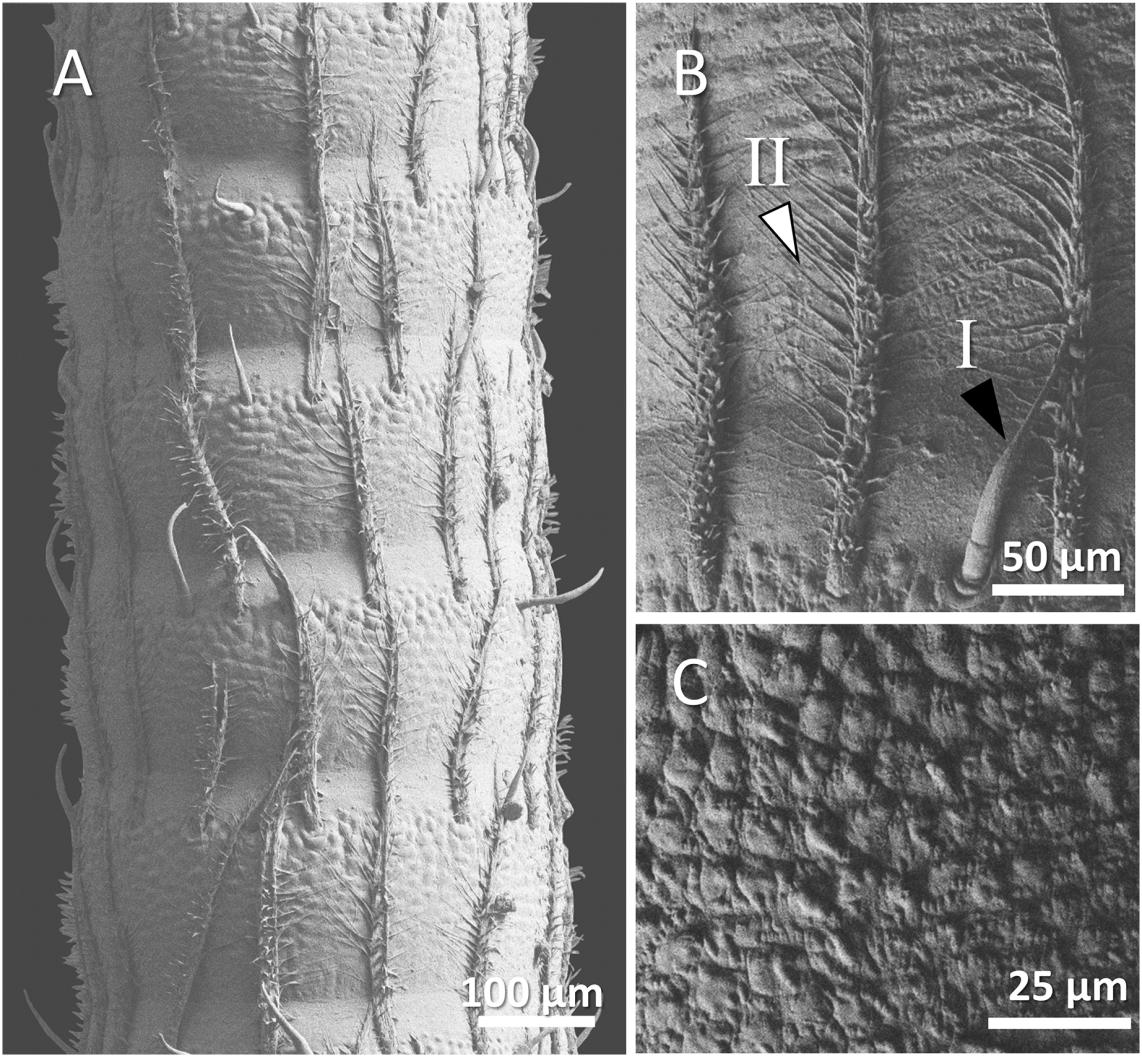
(A) An overview of antenna in common yabby (*Cherax destructor*), (B) a higher magnification of two forms of sensory hair (I, non-branched; II, branched), and (C) a higher magnification of tuberculous surface of antenna.

The scanning electron microscope micrographs showed a general conserved pattern, including segmented organs carrying two forms of sensory hairs on the surface in studied crayfish species (Figs. 3–9). Sensory hairs emerged from the upper part of each segment and extended upwards. While some of the sensory hairs are tubular or cone-shaped (non-branched), the second form consists of feather-like branches which are originated from the main shaft. In addition, there are differences in the ultrastructure of the antenna surface among the studied species (Figs. 3–9).

Mostly sparse and short sensory hairs were observed in the antenna of signal crayfish, and the branched form of sensory hair was sparsely distributed. The surface of the antenna in signal crayfish was squamous (Figs. 4A–4C).

In Mexican dwarf crayfish, we observed mostly the non-branched form of sensory hairs and rarely the branched form. In most cases, the sensory hairs were as long as the length of the next segment. The surface of the antenna in this species is relatively wavy and wrinkled (Figs. 5A–5C).

In marbled crayfish, we observed short and sparse sensory hairs of both forms. The sensory hairs were not frequent and relatively short, and the surface of the antenna is relatively squamous (Figs. 6A–6C).

In red swamp crayfish, both forms of branched and non-branched sensory hairs were visible. They extended distally as far as the middle of the next segment, and the surface of the antenna in this species was relatively smooth (Figs. 7A–7C).

Our observations in spiny-cheek crayfish showed that most of the sensory hairs in this species are branched. The surface of the antenna is relatively squamous (Figs. 8A–8C).

In common yabby, we observed that the frequency of hairs in each segment is higher, and they consist primarily of the long, branched form. The surface of the antenna in this species is not smooth and covered by tubercles. The density of sensory hair is high, and the length of them is extended into half to whole length of an antennal segment (Figs. 9A–9C).

## Discussion

In the present study, we have demonstrated a comparative morphology of the antennae and accompanied sensory hairs among different freshwater crayfish species. The results of our study showed a general conserved pattern of morphology in antennae and their sensory hairs for all studied species. Two forms of sensory hairs were observed on the crayfish antennae, including branched and non-branched types. Similar forms of sensory hairs are supported by the literature. Two morphologically different forms of sensory hairs were detected on the antennae and antennules of narrow-clawed crayfish *Pontastacus leptodactylus* including smooth and feathered sensory hairs. It was shown that while smooth hairs react against the surrounding environment movement and touching, feathered hairs response to direct touch and displacement of main shaft by bending (Bender, Gnatzy & Tautz, 1984; Tautz et al., 1981). It was reported that feathered hairs are innervated by two sensory cells, while smooth hairs could have more than two sensory cells (Tautz et al., 1981). Contrary, it was observed that the feathered hairs in narrow-clawed crayfish (Bender, Gnatzy & Tautz, 1984) and common yabby (Sandeman, 1989) are not innervated and are more likely to serve as structural support for the long and flexible antennae. Also, two forms of beaked and feathered sensory hairs in red swamp crayfish were reported (Mellon, 2012), which are morphologically similar to non-branched and branched hairs in our study. It is possible that these two forms of sensory hairs have two different functions including mechanoreceptors (Kouyama & Shimozawa, 1982) or chemoreceptors (Fedotov, 2009). On the other hand, these could be different developmental stages of a sensory hair. The emergence of a sensory hair from the pore on the surface of the antenna could be facilitated when it is non-branched and then branches appear to increase the surface and sensitivity of sensory hairs to stimuli.

Similar morphology of sensory hairs that were observed on antenna, antennule (Tautz et al., 1981), chelae (Belanger et al., 2008) and maxillipeds (Patoka, Blaha & Kouba, 2017) of crayfish may indicate that these animals use similar sensory appendages with equivalented functions across their bodies.

Antennae and sensory hairs morphology vary among species that may reflect adaptation for necessary mechanosensory and chemosensory requirements in the specific environment (Hallberg, Johansson & Wallen, 1997; Tierney, Thompson & Dunham, 1986). For example, it was stated that surface-dwelling species have a significantly higher density of sensory hairs on their sensory organ compared to cave-dwelling crayfish species, while cave-dwelling species possess longer individual sensory hairs (Ziemba et al., 2003). Also, two cave (troglobitic) crayfish species with well-developed hairs in their maxillipeds, namely *Troglocambarus maclanei* and *Cherax acherontis* probably use their dense setose maxillipeds to collect organic material from the water current as a food source (Patoka, Blaha & Kouba, 2017). In addition, it was shown that different water flow in habitats such as lake, creek, river or hatchery conditions could cause differences in morphological features of sensory hairs in virile crayfish *Faxonius virilis* (Mead, 2008).

Hence, the differences observed in the morphology of sensory hairs among different crayfish species could be caused by different levels of stimuli that they encounter in their specific habitat based on biodiversity, water quality, food availability, the intensity of intra- and interspecific interactions and density of predators. But it is unknown if the differences are evolved by short-term exposures during an individual’s lifetime or by long-term existence in specific habitats/conditions after several generations. Common yabby inhabits dark and turbid waters. Therefore, their tactile senses are a great source of information about their environment that highlights the importance of the antennae and sensory hairs in the common yabby (Basil & Sandeman, 2000). The antennae in common yabby can sense environmental stimuli in the absence of the eye, which is necessary for a dark environment (Varjú, 1989). It could be a reason for their higher density of sensory hairs compared to the other studied species. Also, it could be speculated that tropical species such as common yabby crayfish, which live at sites with higher biodiversity, encounter lots of stimuli and therefore need higher density of sensory hairs. We speculate that species such as signal crayfish that inhabit colder waters with lower biodiversity develop shorter and less dense complements of sensory hairs. However, there is one exception such as kōura crayfish from New Zealand where temperature falls down below 10 °C within the year, and this crayfish is covered by dense hairs (Hopkins, 1967). As a result, different habitats may expose the animals to varying levels of stimuli from the prey, predator, and conspecific, and animals may have to adapt themselves to the environment by changing the morphology and intensity of the sensory hairs, which allows them to fit better in their related environments. It will be promising to compare cultured and wild animals, as well as animals from different localities with different conditions to set up clear explanations for the drivers of the different shapes and densities of crayfish hairs.

Even if we do not know the hidden drivers, we can say that the distribution and quantity of sensory hairs with various functions may have a significant impact on how surrounding information is received and can therefore affect the final response as described in other arthropods such as insects (Dong et al., 2020). It would be interesting to study wild living and cultured animals and the potential differences in the morphology of sensory hairs. The next questions should be then asked about the level of the ability of each species to detect stimuli in the environment in accordance with their density of sensory hairs and morphology. A comparative study showing how different crayfish species react in front of similar stimuli can also help to find a suitable model organism for behavioural and toxicological studies.

## Conclusions

Despite the similar general morphology in the antennae and sensory hairs, there are differences in ultrastructural and biometrical traits that may help in the distinguishment of the studied crayfish species. Two forms of sensory hairs can reflect different functions or different developmental stages of these appendages. The different morphology of antennae might reflect adaptation to the conditions of their specific habitats. Different morphology and density of sensory hairs could also be a mark of different levels of sensitivity to detect chemicals, prey, predators, conspecifics and other stimuli in their environments. Further studies are needed to compare the sensitivity of different crayfish species to the stimuli based on their different sensory hairs density and morphology to find the best model species candidate for the behavioural and toxicological studies.

## Supplemental Information

10.7717/peerj.15006/supp-1 Supplemental Information 1 Data.

## Additional Information and Declarations

### Data Availability

The following information was supplied regarding data availability: The raw data is available in the Supplemental File.
